# The impact of EMTALA on medical malpractice framework models: a review

**DOI:** 10.1186/s13037-022-00325-w

**Published:** 2022-06-24

**Authors:** Amrita Shenoy, Gopinath N. Shenoy, Gayatri G. Shenoy

**Affiliations:** 1 Assistant Professor of Healthcare Administration, University of Baltimore, College of Public Affairs, School of Health and Human Services, Healthcare Administration/Management Program, 1420 N. Charles Street, Baltimore, MD 21201 USA; 2 Medical Malpractice Attorney/Senior Medicolegal Consultant, Post-Graduate Examiner of Law (LLM & PhD) at the University of Mumbai, Former Honorary Professor of Obstetrics/Gynecology at K J Somaiya Medical College and Hospital, Former President and Post-Graduate Examiner of Obstetrics/Gynecology at the College of Physicians and Surgeons of Bombay, and Former Member of the Consumer Disputes Redressal Forum, Mumbai Suburban District, State Government of Maharashtra, Mumbai, India; 3 Former Assistant Professor and Diplomate of the National Board (DNB) Faculty of Anesthesiology, K J Somaiya Medical College and Hospital, Mumbai, Maharashtra India

**Keywords:** Emergency medical treatment and active labor act (EMTALA), Medical malpractice framework models, Defensive medicine model, Physician responsiveness to standard-of-care reforms model, Synergistic visual apparatus, Cascading algorithm

## Abstract

The Emergency Medical Treatment & Active Labor Act (EMTALA) is a healthcare law specific to screening, stabilizing, and transferring (or accepting) patients with emergency medical conditions and active labor. This law, contextual to Medicare-participating hospitals, ensures public access to emergency medical services, regardless of the individual’s ability to pay. The Defensive Medicine (DM) model and Physician Responsiveness to Standard-of-care Reforms (PRSRs) model are two medical malpractice frameworks leveraged in this paper. The nodes of these frameworks comprise of the treatment-versus-no-treatment dynamics and cutoff thresholds. Cutoff thresholds are specific to health risks and treatment price rates. Health risks stem from those with treating or not treating a patient as well as those inherent from the patient’s ailment. Treatment price rates are subcategorized into customary and efficient price rates. Given the above nodes of these frameworks, this paper examines how the above medical malpractice models synchronize and sequentially align with the legal obligations of this law. This paper, furthermore, contemplatively describes how the incentivize/penalize dynamics interrelate to the push/pull dynamics of the PRSRs malpractice model. Thereafter, this paper applies the above push/pull dynamics contextual to the three specific obligations of this law, essentially, screening, stabilizing, and transferring (or accepting) emergency care patients. Conclusively, this paper illustrates the above network in a cascading algorithm that ligates the nodes of these frameworks to EMTALA's obligations.

## Background

In 1986, Congress enacted the Emergency Medical Treatment & Labor Act (EMTALA) [1]. EMTALA (hereinafter referred to as: this act) ensures public access to emergency healthcare services regardless of the ability to pay [1]. The Department of Health and Human Services (DHHS) oversees the enforcement of this act [2].

Enforcement of this act is a complaint driven process [3]. This process spans three agencies that are involved in enforcing this act [2]. Those three agencies are the: (1) Office of Inspector General (OIG); (2) Centers for Medicare & Medicaid Services (CMS); and (3) Office for Civil Rights (OCR) [2, 3]. Both CMS and OIG refer specific cases of this act [2]. CMS oversees the legal and regulatory development process, as part of its general duties towards the Medicare program [1–3].

Sections 1866(a)(1)(I), 1866(a)(1)(N), and 1867 of the Social Security Act (SSA) impose specific obligations of this act on Medicare-participating hospitals [1]. These obligations are screening, stabilizing, and transferring (or accepting) an emergency patient.

First, providing a medical screening examination (MSE) is required when a request is made for an examination or treatment of an emergency medical condition (EMC) [1]. Second, these hospitals are, thereafter, required to stabilize the patient that is diagnosed with an EMC [1]. Third, in the event the hospital is unable to stabilize the patient, it facilitates an appropriate transfer of the patient to another hospital [1].

An extension of the third obligation is that if a Medicare-hospital finds that if an individual has an EMC, it is obligated to provide this individual with either necessary stabilizing treatment or an appropriate transfer to another medical facility where stabilization can occur [1]. In this case, EMCs involve all emergency cases and active labor [1]. This act is particularly applicable to providing MSEs to patients with EMCs, regardless of their ability to pay [1].

A MSE is provided to determine whether or not an underlying medical emergency exists, and if so, this act requires that the hospital, first and foremost, stabilizes the patient [4]. The transferring hospital must completely screen and stabilize the patient given its resources, provide care en route, contact the receiving hospital, and transfer the patient with the appropriate copies of medical records [5]. This act also requires that the receiving hospital should accept patients provided it has the necessary resources to care for such patients [5].

EMTALA is a United States Congressional Act enacted in 1986 as part of the Consolidated Omnibus Budget Reconciliation Act (COBRA) of 1986 [6, 7]. It is commonly referred to as the federal “anti-dumping law” [6, 7]

The above act “provide(s) an ‘adequate first response to a medical crisis for all patients and ‘send(s) a clear signal to the hospital community that all Americans, regardless of wealth or status, should know that a hospital will provide what services it can when they are truly in physical distress” [8, 9]. This act, therefore, requires Medicare hospitals to provide examination and treatment for EMCs and active labor [9].

An EMC is well defined under this act as a medical condition with acute symptoms that would place the health of the patient in serious jeopardy, impair bodily functions, and impair the function of an organ or body part [9, 10].

This act, additionally, defines an EMC with regard to a pregnant woman having contractions within the scope of two aspects [9, 10]. First, inadequate time to result in a safe transfer to another hospital before delivery [9, 10]. Second, that the transfer may pose a threat to the health or safety of the woman or her unborn child [9, 10].

This act, moreover, prevents hospitals from denying or limiting treatment to patients based on their insurance status or ability to pay and transferring them to other hospitals [11]. Failure of hospitals to comply with the obligations of this act can result in stringent penalties [11]. The OIG and CMS impose penalties that range from monetary fines, exclusion from Medicare reimbursement to federal prosecution [11].

The term appropriate medical screening contextual to this act varies on a case-by-case basis [12]. Physicians conducting these MSEs require good clinical judgement to determine whether an emergency exists or not.

If an emergency does exist, it should be treated until stable, unless the patient requires a transfer. A transfer is ideal when medical benefits outweigh risks [12]. This act requires that all patients having EMCs must be treated until stable [12].

This act, furthermore, is exceedingly clear in stating that stabilizing care may not be delayed for the purpose of determining the patient’s “method of payment or insurance status” [13]. Although clear, some ambiguities do exist.

The first ambiguity is knowing when the duty to stabilize occurs [9]. As per this act, it arises when “the hospital determines that the individual has an EMC” [14]. If the patient is in the emergency department of the hospital, it is required of the hospital to conduct the necessary MSE [15]. The second refers to whether or not this act applies only to patients in the emergency department or includes emergencies occurring elsewhere within the hospital premises [9].

This act does not define the term “come(s) to the hospital” but it does define “come to the emergency department”, thereby, requesting examination or treatment in hospital property [16]. The term “hospital property” includes not only the building and grounds but also ambulances owned or operated by the hospital [16]. Persons in non-hospital-owned ambulances on hospital property are also considered to have come to the emergency department [17].

The U.S. Court of Appeals for the Ninth Circuit held that the overarching purpose of this act is to ensure that patients, particularly, the indigent and underinsured, receive adequate emergency medical care [9].

Emergency medical patients are only diverted from the hospital when there is a valid, treatment-related reason for doing so [9]. Furthermore, this act requires hospitals that offer emergency services to screen and stabilize patients within the available infrastructure [9].

This act, furthermore, discourages the practice of “dumping” indigent or uninsured patients. In this regard, it requires that all patients, whether insured, uninsured, or self-pay receive uniform treatment [9].

We consider two medical malpractice framework models which are as follows, the: (1) Defensive Medicine (DM) model; and (2) Physician Responsiveness to Standard-of-care Reforms (PRSRs) model.

This paper aims to explore the pivotal nodes of the above two medical malpractice frameworks, contextual to this act’s three-pronged obligations. Those three being screening, stabilizing, and transferring (or accepting) emergency patients.

This paper has three research questions that stem forth three objectives. First, we describe how the nodes of DM and PRSRs malpractice models interrelate with this act.

Second, we describe how the incentivize/penalize mechanism interrelates with the push/pull dynamics.

Third, we analyze how the push/pull dynamics interrelate to the three obligations of this act.

Finally, we illustrate the interconnections between the above frameworks to this act with an algorithm that visually ligates these research questions.

There are three objectives burgeoning from the above research questions. First, the nodes of the above models aligning with this act would assist better our understanding its interclasping mechanism.

Second, a reasonable understanding of the above alignment would be helpful in foreseeing and averting any prospective malpractice cases.

Finally, this paper’s algorithm would serve as a visual prototype to identify segments for analysts researching further on this topic.

This act has three legal obligations as noted above: (1) providing all emergency patients with MSEs; (2) stabilizing patients that have EMCs; and (3) transferring (or accepting) appropriate emergency patients [1].

The first medical malpractice framework, the DM model is based on the following two pivotal nodes: (1) treatment-versus-no treatment dynamics, and (2) cutoff thresholds based on health risks to the patient [18].

The second medical malpractice framework, the PRSRs model is based on the following two pivotal nodes the: (1) push/pull dynamics of patient care practices, and (2) customary/efficient treatment price rate cutoff [18].

### Defensive medicine model: framework dynamics

Conceptualized by Michael Frakes, the DM model illustrates physician behavior and decision making [18]. The above model captures the relationship between two aspects. First, healthcare spending, and second, contextual to this study, malpractice liability [18]. This model is known as an abstract model of physician decision-making and is based on treatment-versus-no-treatment dynamics [18].

In the first dynamic, the physician can be sued for failing to treat when treatment is required [18]. This dynamic, moreover, applies when the physician exposes a patient to inherent treatment risks that may cause harm in this situation. In the second dynamic, the former can be sued for erroneous processes of care arising from negligent execution of treatment which is separate and distinct from the decision to treat [18].

### Physician responsiveness to standard-of-care reforms model: framework dynamics

The PRSRs model, also conceptualized by Michael Frakes, illustrates price rate cutoffs and liability structure [18]. This model has an inherent liability structure in which liability is assigned to the physician.

Liability (or policy) reform can either push the physician away or pull towards the desired practices of patient care [18]. The above push/pull dynamics is explained with customary and efficient treatment rate cutoffs [18].

### Alignment of EMTALA to DM and PRSRs malpractice framework models

**Fig. 1:**
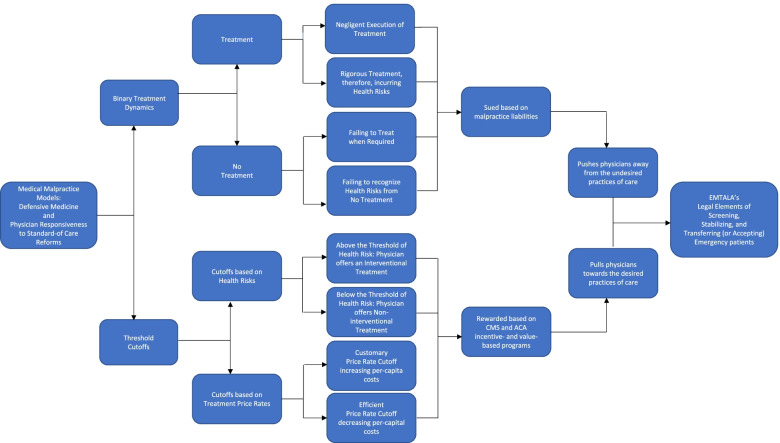
Cascading algorithm representing EMTALA’s interconnection to the above Medical Malpractice Models. [Sources of: (1) The Nodes of the Defensive Medicine and Physician Responsiveness to Standard-of-care Reforms frameworks: Frakes MD. The surprising relevance of medical malpractice law. U. Chi. L. Rev. 2015;82 (1):317–391. Available from: https://www.jstor.org/stable/43234698, and (2) EMTALA’s Legal Provisions from The Centers for Medicare & Medicaid Services. Available from: https://www.cms.gov/Regulations-and-Guidance/Legislation/EMTALA

In Fig. 1, we visually represent the alignment of the DM and PRSRs framework models in synergy with this act. In this process, we illustrate our algorithm with nodular interconnections of both frameworks aligning with this act.

This algorithm, diagrammatically, interconnects the pivotal nodes of both these frameworks with the legal obligations of this act. It assists us in showing how the nodes of these malpractice frameworks align and apply to this act sequentially in a stepwise cascading manner.

The first medical malpractice framework, the DM model is based on the following two pivotal nodes: (1) treatment-versus-no treatment dynamics, and (2) cutoff thresholds based on those determinants [18].

The first node, treatment-versus-no treatment dynamics is binary owing to the dual choice of offering or not offering treatment. This is, furthermore, bifurcated into erroneous processes of care arising from: (1) negligent execution of treatment, and (2) incurring harm from rigorous treatment [18]. The above no-treatment dynamic is, moreover, bifurcated into: (1) failing to treat when treatment is required, and (2) failing to recognize deteriorating health because of no treatment [18].

The second node, cutoff threshold, is categorized into health risk cutoffs and treatment price rate cutoffs [18]. Health risk cutoffs are based on clinical determinants. Treatment price rate cutoffs are based on externally induced factors such as a court-based order or policy reform [18].

Health risk cutoffs are, furthermore, bifurcated into risks that are mostly above or below a threshold [18]. This threshold is based on the intensity of health risks borne by the patient [18]. A health risk cutoff that separates an interventional from a non-interventional treatment option depends on the intensity of health risk factors [18].

Health risk factors play a critical role in determining what type of treatment is required [18]. If risk factor levels are above this cutoff, the surgeon may decide to opt for treatment that is interventional in nature. If risk factor levels are below this cutoff, he or she may decide to opt for treatment that is non-interventional in nature, and therefore, consider other diagnostic and case management options.

Surgeons offer a choice of an interventional coronary artery bypass graft or a clinically managed non-interventional treatment plan for patients with cardiac anomalies according to prevailing health risk factors, as an example.

Clinical determinants are those factors that influence treatment cutoffs, examples of which are, clinical training, incentives, and motivation to procure patient health outcomes, etc. [18]. These are examples of determinants that influence a physician’s decision-making process for electing treatments. There are additional risks to the patient’s health arising inherently from the patient’s ailment.

These risks become more severe as complications in both health and treatment increase [18]. If the patient is left medically untreated, consequences from failing to treat may be potentially severe. If the patient is rigorously treated despite inherent health risks, consequences from this action may, furthermore, harm the patient. This situation, therefore, compounds because of the risks incurred from treatment and those inherent from the condition itself.

The second medical malpractice framework, the PRSRs model is based on the following two nodes, the: (1) push/pull dynamics towards the practices of patient care; and (2) treatment price rate cutoffs [18]. Acts or laws that are statutory, court-based, or policy reforms are examples of external or environmental factors contextual to this model [18].

The first node, the push/pull dynamics, explains how physicians are potentially pushed away from and pulled towards the required practices of patient care [18]. The second node, treatment price rate cutoff is categorized into: (1) customary price rate cutoff, and (2) efficient price rate cutoff [18].

An example policy reform here is the conversion from fee-for-service reimbursement to capitation. Fee-for-service reimbursement is based on the volume of services provided whereas capitation is based on value.

There is a likelihood that adopting volume-based fee-for-service reimbursement would induce physicians to follow their customary price rate cutoffs. Adopting the value-based capitated system, conversely, would induce physicians to follow the efficient price rate cutoff.

Following the customary price rate cutoff is one that has propensity to increase healthcare spending. Following an efficient price rate cutoff, on the contrary, has propensity to decrease excessive healthcare spending.

Replacing the efficient price rate cutoff with the customary one would incline physicians to elect treatments in which treatment options would either be cost-beneficial or budget-neutral [18]. The motivation, here, would be to decrease healthcare spending. Physicians would be pushed from volume-based towards value-based patient care practices.

The passage of the 2010 Affordable Care Act (ACA), in this landscape, implemented the capitated value-based reimbursement system. The ACA, furthermore, implemented multiple programs to incentivize physicians based on adding value and improving population health.

The Merit-based Incentive Payment System, Hospital-Acquired Condition Reduction Program, Hospital Readmissions Reduction Program, the shared-savings based Accountable Care Organizations, Hospital Inpatient Quality Reporting Program, and Hospital Outpatient Quality Reporting Program are examples of CMS and ACA based physician-incentivized programs.

Physicians are incentivized for carefully evaluating treatments and participating in ACA policy programs that support value-based capitation [18]. Physicians can be sued, as well, for untoward consequences arising from treatment-versus-no treatment dynamics [18].

On the one hand, being sued for the untoward consequences of patient care may serve as an impetus to push physicians away from the undesired practices of patient care. On the other hand, being incentivized for participating in ACA-based programs may serve as an impetus to pull physicians towards the required practices of patient care.

In the context of this act, physicians and/or hospitals are penalized for not abiding by the legal obligations of this act. A safeguarding avenue would be to screen, stabilize, and transfer (or accept) emergency patients in applicable cases.

In the medicolegal landscape, given the push/pull dynamics, penalties based on malpractice liabilities would push physicians away from the undesired consequences of this act, and concurrently, pull physicians towards the required practices of patient care.

In the first step, the physician performs a MSE to detect whether the patient has an EMC. If so, the second step would be to stabilize the patient. The final step would be to transfer the patient, if applicable. If the hospital is not equipped with the necessary infrastructure to stabilize or treat the patient, then it facilitates transfer of the patient to the receiving hospital that is equipped with those necessary resources.

Given this review’s limitations, first, it considers only two medical malpractice frameworks, DM and PRSRs models. Second, it illustrates the alignment of the above frameworks relative only to this act. Third, it is selectively specific to this act’s tri-pronged obligations. Fourth, it projects the alignment of this act to the pivotal nodes of the above framework models. Fifth, it jointly analyzes the above framework models with this act in a cascading algorithm. Finally, this study is limited only to this act’s obligations of screening, stabilizing, and transferring (or accepting) emergency patients.

Despite the above limitations, this review has some strengths. First, this study’s algorithm visually represents how these frameworks interconnect, and thereafter, align with this act. Second, a thorough study of the existing literature failed to detect a paper that visually and descriptively explained the above alignment contextual to this act. Third, this review’s visual and descriptive explanation would further assist analysts to better place specific clinical practices within this algorithm. A likely benefit of the above placement would be to bolster compliance towards this act. A close advantage would be foreseeing and averting any deviations from this act.

## Conclusion

The purpose of this paper was to examine the above medical malpractice frameworks that align with this act through three analytical spectra. In the first spectrum of our analysis, we observe how the nodes of the DM and PRSRs models align with this act. In the second spectrum, we describe how the incentivize/penalize mechanism aligns with the push/pull dynamics. In the third spectrum, we note how the push/pull dynamics interconnect with the provisions of this act. We visualize these interconnections with a cascading algorithm to depict our observations. In general, the law making procedure entails three important steps. First, a Bill is drafted after society recognizes the need for a law based on a public wrong. This Bill is put in the public domain for feedback and comments. Second, this Bill is, thereafter, placed in both the House of Representatives and Senate, after incorporating applicable comments. Once this bill passes both the House and Senate, with or without further modifications, it then becomes an Act. An Act, by itself, is not enforceable at this stage. Third, once the President signs it and once it is gazetted, this Act then becomes an enforceable Law or an enforceable Statute. A Statute and Law are terms that are one and the same. A Statute is legal term and a Law is a layman's term. Encapsulating this process, EMTALA underwent numerous revisions since August 1986 and continues to do so. The word Active was eventually omitted. As of March 9, 2020, CMS issued a memorandum to address EMTALA requirements and implications specific to this ongoing continually mutating global COVID-19 pandemic. Within the encompassing scope of this review, this paper visually ligates the nodes of the above malpractice frameworks to EMTALA's legal obligations in a stepwise sequentially cascading algorithm.

## Data Availability

Not applicable.

## Abbreviations

ACA: Affordable Care Act
CMS: Centers for Medicare & Medicaid Services
COBRA: Consolidated Omnibus Budget Reconciliation Act
DHHS: Department of Health and Human Services
DM: Defensive Medicine
EMC: Emergency Medical Condition
EMTALA: Emergency Medical Treatment and Active Labor Act
MSE: Medical Screening Examination
OCR: Office for Civil Rights
OIG: Office of Inspector General
PRSRs: Physician Responsiveness to Standard-of-Care Reforms
